# Novel approaches to identify protective malaria vaccine candidates

**DOI:** 10.3389/fmicb.2014.00586

**Published:** 2014-11-17

**Authors:** Wan Ni Chia, Yun Shan Goh, Laurent Rénia

**Affiliations:** ^1^Singapore Immunology Network, Agency for Science, Technology and ResearchSingapore, Singapore; ^2^Department of Microbiology, Yong Loo Lin School of Medicine, National University of SingaporeSingapore, Singapore

**Keywords:** malaria, vaccine, library, antibodies, screen

## Abstract

Efforts to develop vaccines against malaria have been the focus of substantial research activities for decades. Several categories of candidate vaccines are currently being developed for protection against malaria, based on antigens corresponding to the pre-erythrocytic, blood stage, or sexual stages of the parasite. Long lasting sterile protection from *Plasmodium falciparum* sporozoite challenge has been observed in human following vaccination with whole parasite formulations, clearly demonstrating that a protective immune response targeting predominantly the pre-erythrocytic stages can develop against malaria. However, most of vaccine candidates currently being investigated, which are mostly subunits vaccines, have not been able to induce substantial (>50%) protection thus far. This is due to the fact that the antigens responsible for protection against the different parasite stages are still yet to be known and relevant correlates of protection have remained elusive. For a vaccine to be developed in a timely manner, novel approaches are required. In this article, we review the novel approaches that have been developed to identify the antigens for the development of an effective malaria vaccine.

## INTRODUCTION

Malaria is an infectious disease caused by the protozoan parasite *Plasmodium* and transmitted by the *Anopheles* mosquitoes. Malaria is a major public health problem, leading to high mortality and morbidity. Nearly half the world’s population is at risk of contracting malaria (CDC, 2012). There are 207 million cases of clinical malaria and approximately 627,000 deaths in WHO (2012). There is currently no available vaccine. Age and host immune status are high risk factors for malaria, with young children under the age of five, pregnant women and travelers or migrants who lack immunity to the disease being most susceptible. Other risk factors include the infectivity and the transmission dynamics of the parasite strain (Doolan, 2011).

The *Plasmodium* parasite has a complex life cycle. Following an infected mosquito bite, sporozoites are inoculated into the dermis of the mammalian host (Vanderberg and Frevert, 2004; Amino et al., 2006). The sporozoites travel to the liver *via* the bloodstream and infect the hepatocytes (Amino et al., 2006). During this phase in the hepatocytes, sporozoites develop into schizonts over 2–14 days, depending on the species. Merosomes, merozoites containing vesicles, eventually bud out from infected hepatocytes to release merozoites, which then infect erythrocytes (Sturm et al., 2006; Baer et al., 2007). Some of the blood stage parasites undergo sexual differentiation into male and female gametocytes that can be taken up by a feeding Anopheline during a blood meal. Ookinetes, which results from gametocyte fusion, develop into oocysts in the midgut of the mosquito. Upon oocyst maturation, newly formed sporozoites migrate to the salivary gland of the mosquito, awaiting the next blood meal (Moorthy et al., 2004).

Symptoms of malaria include fever, headache, chills, sweating, and vomiting. Recurrent fever is one of the hallmarks of clinical malaria. This is a consequence of the release of malarial toxins into the bloodstream following repetitive rupture and re-invasion of erythrocytes. With disease progression, the red blood cell counts decreases and severe anemia might occur. Malarial infected red blood cells, such as those of *Plasmodium falciparum*, can also sequester in deep tissues, causing cerebral malaria, and organ failure. These severe pathologies can eventually lead to death.

## IMMUNE RESPONSES TO A MALARIA INFECTION

Protective immunity against malaria requires a timely and coordinated interplay between the innate and adaptive immunity. This involves dendritic cells, NK cells, B cells, CD4^+^ and CD8^+^ T cells (Stevenson and Riley, 2004).

Sporozoite-specific antibodies can block sporozoites from migrating to the liver or from invading into hepatocytes, arresting disease progression (Rathore et al., 2005; Finney et al., 2014). Antibody-mediated immunity has been thought to be the central effectors of parasite clearance in the peripheral blood as MHC class I/II molecules are absent on the surface of infected red blood cells (Langhorne et al., 2008). The importance of antibodies was first demonstrated by Cohen et al. (1961), showing that passive transfer of immunoglobulins from immune adults into naïve, infected children resulted in rapid reductions of parasite density and resolution of clinical symptoms (Cohen et al., 1961). Merozoite-specific antibodies can prevent merozoites from invading erythrocytes (Michon et al., 2000; Dutta et al., 2005; Jiang et al., 2011) and mediate clearance of infected red blood cells by phagocytic cells *via* antibody-dependent cellular inhibition (Marsh and Kinyanjui, 2006). Pathogen-specific antibodies secreted by B cells with CD4^+^ T helper cells enhancement are essential for clearance of parasitemia in the later stages of the infection (Langhorne et al., 2008).

In addition to the humoral arm of the adaptive immunity, cell-mediated immune responses are also crucial for protection against malaria. CD8^+^ and CD4^+^ T cells kill infected hepatocytes through diverse mechanisms (Renia et al., 1993; Doolan and Hoffman, 2000; Frevert et al., 2009; Trimnell et al., 2009; Cockburn et al., 2013) and induce sterile protection (i.e., no blood stage infection) in mouse models. Recent work has revealed an important role for IFNγ-secreting CD8^+^ T cells in preventing chronic *P. chabaudi* blood stage infection in mice (Horne-Debets et al., 2013). In human, sterile protection has been observed in experimental sporozoite challenge experiments following vaccination with whole sporozoites (Hoffman et al., 2002; Roestenberg et al., 2009; Seder et al., 2013). Both sporozoite-specific antibodies and T cells were induced.

## VACCINE DEVELOPMENT AGAINST MALARIA

The rationale for vaccine development to protect against malaria stems from observations where naturally acquired immunity to malaria can protect individuals living in malaria-endemic regions against malaria in an age-dependent and exposure-dependent manner (Gupta et al., 1999; Schofield and Mueller, 2006; Crompton et al., 2010). Although the protection is not sterilizing and is not always ensured for all chronically exposed individuals, passive transfer of sera from some chronically exposed individuals reduced strongly parasite levels in infected individuals (Cohen et al., 1961; Bouharoun-Tayoun et al., 1995). This demonstrated that antibodies can offer protection against the blood phase of the malarial infection.

More rationally, the typical Pasteur approach where attenuated parasites were used as vaccines has further demonstrated the feasibility of vaccination as protection against malaria. Immunization with irradiation-attenuated sporozoites has shown to confer sterile immunity against sporozoites challenge in animal models and in humans (Richards, 1966; Nussenzweig et al., 1967; Clyde et al., 1973; Rieckmann et al., 1974; Hoffman et al., 2002). The protective immune response involving antibodies and T cells was shown to target the pre-erythrocytic stages (Nussenzweig et al., 1967; Overstreet et al., 2008). In recent years, immunization with sporozoite or blood parasite under drug cover was also shown to confer strong protective immunity involving antibodies and T cells (Belnoue et al., 2002; Renia et al., 2006; Roestenberg et al., 2009; Friesen et al., 2010). Other approaches have used genetically attenuated parasites. Although these approaches have led to sterile immunity in mice (Mueller et al., 2005; Butler et al., 2012), they have yet to prove their efficacy in humans (Spring et al., 2013).

After decades of efforts spent on developing vaccines against malaria, no strong vaccine candidate has emerged as yet. Most vaccine development efforts are focused on only four antigens (WHO, 2012). The most clinically advanced candidate, RTS,S, conferred ~50% protection from clinical *P. falciparum* malaria in children aged 5–17 months, and ~30% protection in children aged 6–12 weeks (Agnandji et al., 2011, 2012). Vaccine efficacy was undetectable 3 years after vaccination (Bejon et al., 2013). This level of protection is suboptimal and too low to achieve malaria eradication. No single molecular signature, key cellular determinant or immune mechanism of naturally acquired or vaccine-induced immunity has been unequivocally associated with protection (Offeddu et al., 2012). This impediment toward vaccine development for malaria is multi-factorial. The *Plasmodium* parasite has a diverse protein repertoire, and a complex life cycle involving both vertebrate and invertebrate hosts. There are antigenic polymorphisms and variations across parasite strains and species, and the parasite has developed sophisticated strategies to evade the host immunity.

With more reports of drug resistance and insecticide resistance in some endemic regions (Trape et al., 2011; Phyo et al., 2012; Ashley et al., 2014), management of the malarial disease has been increasingly difficult. Identification of new malarial antigens for vaccine development is critical. Here, we reviewed the different techniques that have been used to identify antigens recognized by antibodies induced during natural infections or after vaccinations with whole parasites.

## PRE-GENOMIC SCREENING METHODS FOR PROTECTIVE ANTIGENS

The advent of new molecular biology techniques in the 1980s led to the discovery of many *Plasmodium* antigens. DNA libraries, consisting of cDNA or genomic DNA, were constructed in bacteriophage vectors for expression in *Escherichia coli* (Kemp et al., 1986). Antigens such as the circumsporozoite protein (CSP), the S-antigen, the ring erythrocyte surface antigen (RESA) were the first to be identified using mouse monoclonal antibodies (Ellis et al., 1983), sera from infected humans (Kemp et al., 1983; Stahl et al., 1984) or sera from infected mice or monkeys (Anders et al., 1984; Brown et al., 1984; Ardeshir et al., 1985). All the antigens discovered using these approaches were immunodominant or possessed immunodominant regions made of repeats (Kemp et al., 1987). Although immunodominant antigens induce very strong antibody responses, recent studies have shown that they did not offer adequate protection when tested as subunit vaccines in clinical trials (Spring et al., 2009; Doolan, 2011; Sheehy et al., 2012), suggesting that these antigens might be used by the *Plasmodium* parasite for immune evasion (Anders, 1986; Rodriguez et al., 2008). The CSP, an antigen of pre-erythrocytic stage malaria and the first malaria antigen to be cloned (Ellis et al., 1983), is one such example. Despite being immunodominant, the Phase III vaccine efficacy was suboptimal, with 55% reduction in the frequency of malaria episodes during the 12 months of follow-up in children 5–17 months of age and 35% in children 6–12 weeks old at first immunization (Agnandji et al., 2011). The protection waned to a mere 16.8% after 4 years of follow-up, indicating the lack of long lasting protection which declined with time and exposure to the parasite (Olotu et al., 2013). Similarly, while immunization with the apical membrane antigen-1 (AMA-1), an antigen expressed both at the pre-erythrocytic and erythrocytic stages and strongly immunodominant during the erythrocytic stage, elicited functional antibodies with *in vitro* inhibition against homologous blood parasites, volunteers developed parasitemia upon challenge with controlled human malaria infection (infection by mosquito bites) without a significant reduction of peak parasitemia nor delay to patency (Spring et al., 2009).

The cDNA library approach is advantageous with a good representation of a stage-specific *Plasmodium* proteome, depending on the stage of the parasite at which the RNA was extracted from. However, the repertoire of the *Plasmodium* proteome the cDNA library represents is not entirely comprehensive and does not include antigens expressed by the parasite in the other stages of its life cycle. Nevertheless, it has successfully identified more than a hundred malaria antigens. Recently, Raj et al. (2014) created a *P. falciparum* blood stage cDNA library constructed in bacteriophage and expressed in *E. coli*. The antigen library was screened against sera from either infection-resistant or – susceptible chronically exposed individuals living in malaria-endemic regions. 2 out of 3 antigens identified were novel antigens. Antibody to one of the identified novel antigens, PfSEA-1, inhibited parasite growth and blocked schizont egress. Using the *P. berghei* ANKA strain ortholog of PfSEA-1, the authors demonstrated reduced parasitemia and longer survival in mice vaccinated with PbSEA-1. They further validated PfSEA-1 as a promising vaccine candidate through epidemiological studies, where anti-PfSEA-1 antibodies were strongly associated with lesser incidence of severe malaria in Tanzanian children and lower level of parasitemia in Kenyan adults.

## SCREENING METHODS TO IDENTIFY NEW ANTIGENS IN THE ERA FOLLOWING GENOME SEQUENCING

At the turn of the 20th century, whole genomes of many *Plasmodium* strains were sequenced. These genome sequencing initiatives, by The Wellcome Trust Sanger Institute and The Institute for Genomic Research, have provided a wealth of data for the identification of protective antigens (Carlton et al., 2002; Gardner et al., 2002). In this review, we discuss the novel approaches (summarized in **Table 1**) to screen libraries of malarial antigen using sera from protected versus unprotected individuals to identify antigens associated with protection, in search for new protective antigens for vaccine development against malaria.

**Table 1:** Approaches taken to identify protective malarial antigens for vaccine development.

	Type of antigen library	Antigen expression system	Size of library	Type of screening	Sera/antigens that library is screened against	Reference
Pre-genomic era	cDNA	*E. coli* cell-based		*In vitro* immunoscreen	Mouse monoclonal antibodies	Ellis et al. (1983)
	cDNA	*E. coli* cell-based	10 000 clones	*In vitro* immunoscreen	Sera from infected human individuals	Kemp et al. (1983), Brown et al. (1984), Stahl et al. (1984)
	cDNA	*E. coli* cell-based	10 000 clones 10 000 clones 9 000 clones	*In vitro* immunoscreen	Sera from infected mice, mice and rabbits, monkeys	Brown et al. (1984) Anders et al. (1984) Ardeshir et al. (1985)
Post-genomic era	cDNA	*E. coli* cell-based	1 250 000 clones	*In vitro* immunoscreen	Sera from infection-resistant and –susceptible chronically exposed human individuals	Raj et al. (2014)
	Recombinant proteins	*E. coli* cell-free *in vitro* translation	250 antigens	*In vitro* immunoscreen	Sera from protected and non-protected individuals following vaccination with radiation-attenuated sporozoites	Doolan et al. (2008)
	Recombinant proteins	*E. coli* cell-based and wheat germ cell-free system	46 antigens	*In vitro* immunoscreen	Sera from malaria-exposed children	Richards et al. (2013)
	Antigens expressed on cell surface	Mammalian cell-based	80 antigens	*In vitro* immunoscreen	Sera from protected and non-protected individuals following vaccination with live sporozoites	Chia et al. (2014)
	Whole parasite lysates	*P. yoelii* parasite	Whole proteome	*In vitro* immunoscreen	Affinity-purified IgG from immune mice that naturally survived a lethal infection	Kamali et al. (2012)
	DNA (exons)	–	19 genes	*In vivo* screen in mice for protection	–	Haddad et al. (2004)
	Recombinant proteins	Mammalian cell-based	51 antigens	*In vitro* protein–protein interaction screen	Malarial antigen, PfRh5	Crosnier et al. (2011)

## NEW APPROACHES TO SEARCH FOR ANTIGENS INVOLVED IN PROTECTION

The availability of whole genome sequences has enhanced the understanding of the parasite biology. Protein functions can be predicted and validated. Potential antigens could be screened for specific characteristics such as surface expression. Comparative genomics between different parasite strains allows identification of antigens that have limited variation, with the promise of greater vaccine coverage and hence efficacy in the field (Kooij et al., 2005).

Transcriptomics and proteomics has provided critical information on the expression profiles of malarial proteins during the parasite’s life cycle (Florens et al., 2002; Le Roch et al., 2002; Bozdech et al., 2003; Hall et al., 2005) – the mosquito stage (Lindner et al., 2013), the liver stage (Tarun et al., 2008), and the sexual stage (Lasonder et al., 2002; Khan et al., 2005). *In silico* analysis of stage-specific transcription pattern identifies antigens that are differentially expressed for specific targeting to a specific stage of the parasite’s life cycle and also antigens with conserved expression across the different stages for cross-stages targeting (Florens et al., 2002).

### SCREENING RECOMBINANT PROTEIN MICROARRAYS AGAINST IMMUNE SERA FROM VACCINATED HUMAN INDIVIDUALS

Using the published *P. falciparum* and *P. vivax* genomes, a set of selected genes was targeted for protein expression using an *in vitro* transcription/translation system (Doolan et al., 2008; Tsuboi et al., 2008; Crompton et al., 2010; Barry et al., 2011; Trieu et al., 2011; Molina et al., 2012; Baum et al., 2013; Lu et al., 2014). These recombinant proteins were then printed onto microarray chips and probed with immune sera that were obtained from human volunteers naturally exposed to malaria infections or immunized with radiation-attenuated sporozoites. Doolan et al. (2008) generated a protein microarray with 250 *P. falciparum* proteins and used it to profile antibody responses in sera from four groups of individuals: (1) protected, (2) non-protected individuals following vaccination with radiation-attenuated sporozoites, (3) partially protected individuals due to natural exposure, and (4) non-exposed individuals. The same group then expanded their library to include 1204 *P. falciparum* proteins (representing 23% of the *P. falciparum* genome; Crompton et al., 2010; Trieu et al., 2011; Baum et al., 2013) and also 91 *P. vivax* proteins (Molina et al., 2012). 22–29% of the screened proteins were found to be serodominant in immune sera, demonstrating a broad and varied response to many antigens. Antibody titres against current vaccine candidates such as CSP, atypical membrane antigen-1, liver stage antigen-3, merozoite surface protein-1 did not differ between immune and non-immune individuals (Crompton et al., 2010; Trieu et al., 2011).

Richards et al. (2013) expressed *P. falciparum* merozoite proteins, reported to have a role in erythrocyte invasion and/or localized on the merozoite surface or in the invasion organelles of the merozoites, in either bacterial or wheat germ cell-free expression systems. The recombinant proteins were tested for IgG immunoreactivity using sera from malaria-exposed Papua New Guinea children in ELISA. Forty six proteins were selected based on their ability to coat ELISA plates and their immunoreactivity. Sera from older children had higher immunoreactivity to most of the 46 proteins as compared to the younger children, indicating an acquisition of antibody responses with age, presumably due to prolonged exposure to the parasite. While merozoite surface proteins had higher seropositivity compared to the rhoptry and micronemal proteins, the antigens strongly correlated with protection were the rhoptry and micronemal proteins. This is consistent with other studies, suggesting that the key protective malarial antigens are the non-immunodominant antigens (Doolan et al., 2003, 2008; Crompton et al., 2010; Trieu et al., 2011; Baum et al., 2013). The authors proposed that a combinational vaccine consisting of non-immunodominant antigens, such as EBA, PfRh2 and PfRh4, is more likely to offer greater protection (Richards et al., 2013).

One of the main drawbacks with protein array is the extensive efforts needed to generate the soluble recombinant malarial antigens in the library (Doolan et al., 2008; Tsuboi et al., 2008). Cell-based expression systems have met with many difficulties. *P. falciparum* genes have a high A/T content and a substantial number of the genes encode stretches of repeat sequences (Gardner et al., 2002), hindering protein expression in cell-based expression system. Only 30% out of 1000 genes investigated by Mehlin et al. (2006) can be expressed in *E. coli* and a mere 6.3% of the proteins are soluble. The alternative is the wheat germ cell-free expression system. Tsuboi et al. (2008) were able to express 93 out of 124 *P. falciparum* genes as soluble proteins using the wheat germ cell-free system. Rui et al. (2011) reported greater immunogenicity of wheat germ proteins (as opposed to identical proteins produced in *E. coli*) in mice. The wheat germ cell-free expression system could be more suitable for producing antigens for vaccine development.

### SCREENING CELL-ASSOCIATED ANTIGEN LIBRARIES AGAINST IMMUNE MOUSE SERA

We recently cloned a library of 80 malarial antigens into a mammalian surface expression vector pDisplay (Invitrogen; Chia et al., 2014). They transfected these expression vectors into mammalian cells and the antigens were expressed on the cell surface, creating a library of antigen-presenting cells. The library was screened against immune and non-immune sera obtained from mice immunized with live sporozoites or blood parasites under drug cover (Belnoue et al., 2008). Similarly, all immunized volunteers in the study were protected from sporozoites challenge. The antibody repertoire of immunized volunteers was extremely broad and varied. MAEBL was found to be strongly associated with protection. MAEBL has been implicated in invasion into hepatocytes by sporozoites and merozoites into erythrocytes (Kappe et al., 1998, 2001). It was previously demonstrated that anti-MAEBL antibodies to inhibit *P. yoelii* sporozoites invasion *in vitro* in primary mouse hepatocytes, suggesting that MAEBL is a promising attractive vaccine candidate (Singh et al., 2004).

This approach removes the difficulty of expressing and purifying antigens as soluble recombinant proteins. However, the library size in this study represents a small representation of the entire *Plasmodium* genome (~1%) and antigens screened were mainly restricted to those with reasonably good transfection efficiency.

### SCREENING WHOLE PARASITE LYSATES AGAINST AFFINITY-PURIFIED IgG FROM NATURALLY SURVIVING MICE

Due to ethical and technical restrictions, identification of protective immune correlates in humans can only be evaluated in the peripheral blood component. This hinders the study of a vital stage of the parasite’s life cycle, the liver stage. It also limits the examination of the host immune responses in the secondary lymphoid organs such as the spleen, which are also important sites for the induction of protective immunity.

The use of comparative genomic analysis, through the construction of genome-wide synteny maps, has identified other non-human models of malaria (Carlton et al., 2002; Tachibana et al., 2012). Mouse models have played a vital role in the understanding of the immunobiology of malarial infections. The mouse malaria parasite, *P. yoelii*, has been used as a model for malaria research due to the substantial similarity to the human parasite *P. falciparum* (Hall et al., 2002). In addition to being cheaper to maintain and easier to handle, the mouse also has a well characterized immune system, hence offering great advantages over non-human primate models (Wykes and Good, 2009; Taylor-Robinson, 2010). Infection studies of rodent malaria species and their hosts have provided information on parasite biology and pathogenicity. Identification of orthologs allows preclinical validation of new chemotherapies and vaccine candidates. The lethal *P. yoelii* model, which causes death in BALB/c mice, is an excellent model for investigating the vaccine efficacy of the candidates *in vivo* (Langhorne, 1994). Kamali et al. (2012) observed that 20% of ICR mice infected with the lethal strain of *P. yoelii* cleared the infection and these naturally surviving mice had boosted immunity following a second challenge. They affinity-purified the IgG from these mice and probed against whole parasite lysates. Using MALDI-TOF analysis, the antigenic specificities of the protective IgG were Heat shock protein 70 (HSP70), protein disulphide isomerase, plasmepsin and a 39 kDa subunit of eukaryotic translation initiation factor 3. Although the authors did not validate the protective potential of the identified antigens, the approach used in the study identified novel antigens (with the exception of PfHSP70.1 (Haddad et al., 2004). Antibodies against PfHSP70.1 have been shown to eliminate *in vitro* liver stage parasites through antibody-dependent cell-mediated cytotoxic mechanisms (Renia et al., 1990).

### SCREENING FOR PROTECTIVE DNA VACCINE CANDIDATE IN MOUSE CHALLENGE MODEL

New molecular technologies such as Gateway cloning make it easy to clone large numbers of genes into plasmids. Using this technology, Haddad et al. (2004) produced an expression library of 182 *Plasmodium* exons coding for pre-erythrocytic antigens in the mammalian immunization vector VR1012. They immunized mice with 19 out of the 182 cloned vectors, either singly or in combination, and assessed vaccine efficacy following sporozoites challenge by examining the ability of the immunized mice to reduce liver stage parasite load. The most promising DNA vaccine candidate identified was Py01316, annotated as a Qa-SNARE protein in PlasmoDB, which gave a 68–79% reduction in parasite load.

The approach taken by Haddad et al. (2004) has the benefit of generating a library of DNA vaccine candidates, targeting many *Plasmodium* genes with great ease, and does not require the laborious process of generating recombinant proteins. It also provides information on the immunogenicity of the DNA constructs and *in vivo* effectiveness of the induced immune responses to reduce parasite load. However, screening for protective DNA vaccine targets in mice is laborious and requires a large number of mice. As a result, the authors screened a total of 19 out of the 182 cloned plasmids, which limited the number of malarial antigens that can be validated for protective efficacy.

### TARGETED SCREENING TO IDENTIFY MALARIAL ANTIGENS AND THEIR RECEPTORS

Crosnier et al. (2011) expressed a library of erythrocyte surface proteins with the mammalian expression system. Using the avidity-based extracellular interaction screen (AVEXIS), they screened recombinant PfRh5 protein (the bait protein), which is critical for erythrocyte invasion by merozoites, against the library of erythrocyte surface proteins (the prey protein). The PfRh5 protein was found to interact with only one erythrocyte surface protein, basin. The anti-basin monoclonal antibodies were highly effective at blocking *P. falciparum* invasion into erythrocytes *ex vivo* and these blocking effects were efficacious with 15 other culture-adapted and field strains of *P. falciparum*. These data have implications for novel therapeutics. While this does not directly identify new protective malarial antigens for vaccine development, the identification of basin as the binding partner of PfRh5 and perhaps further characterisation studies on the expression levels of basin by the target population would provide critical information on the vaccine efficacy of PfRH5, should PfRh5 be developed as a vaccine candidate. Understanding how basigin binds to PfRh5 could also facilitate rational design of drug compounds to block *P. falciparum*’s invasion into erythrocytes.

One of the shortcomings of AVEXIS is the generation of the library of recombinant proteins, which is laborious. This approach also requires the prior identification of a known bait protein, which is difficult for identifying novel host–parasite interacting antigen pairs. However, the AVEXIS assay is advantageous in its ability to detect direct low-affinity protein interactions, which might not be detectable in other screening methods. More recently, this type of library has shown to be a powerful tool to identify new protective antigens for potential vaccine target development. Using sera from a longitudinal study in a cohort of Kenyan children, Osier et al. (2014) identified 10 antigens (PF3D7_1136200, MSP2, RhopH3, P41, MSP11, MSP3, PF3D7_0606800, AMA1, Pf113, and MSRP1) that were associated with protection against clinical episodes of malaria. While the AVEXIS approach has been used to identify blood stage antigens in this study, the study could be extended to include antigens for other stages.

### IDENTIFICATION OF PROTECTIVE PRE-ERYTHROCYTIC ANTIGENS

Pre-erythrocytic antigens have been attractive targets for vaccine development. This is mainly due to the demonstration that sterile immunity against malaria is achievable in experimental sporozoite challenge experiments in humans following vaccination with whole sporozoites (Hoffman et al., 2002; Roestenberg et al., 2009; Seder et al., 2013).

Most of the above described approaches for identification of protective antigens have used sera from chronically exposed individuals living in endemic regions to screen for protective malarial antigens. Although the approaches have identified mainly blood stage antigens, some of the identified blood stage antigens, such as AMA1 (Silvie et al., 2004), TRAP (Robson et al., 1995), EBA175 (Gruner et al., 2001b), PfEMP3 (Gruner et al., 2001a), and HSP70.1 (Renia et al., 1991), which are also expressed during the liver stage.

To screen for protective pre-erythrocytic antigens, it is essential to have the appropriate sera sets against which the library of malarial antigens will be screened. While sera from chronically exposed individuals living in endemic regions are relatively easier to obtain, they are more likely to have an antibody repertoire that is predominantly specific for blood stage antigens, hence might not be suitable for the identification of liver stage antigens. Marchand and Druilhe (1990) and Gruner et al. (2003) were the first to show in human that chloroquine prophylaxis protected against blood stage infection and use the protected sera to screen for protective antigens. This led to the identification of liver-specific (LSA1; Guerin-Marchand et al., 1987) or cross stage-specific antigens stages (LSA-3, STARP, and SALSA; Fidock et al., 1994; Bottius et al., 1996; Daubersies et al., 2000). Recently, the studies by Trieu et al. (2011) and Chia et al. (2014) involved the use of sera from animals immunized with sporozoites. Trieu et al. (2011) identified 16 previously uncharacterized pre-erythrocytic antigens (Trieu et al., 2011), while the pre-erythrocytic MAEBL antigen was identified by Chia et al. (2014).

## CONCLUSION

Historically, vaccine development efforts have been focused on immunodominant antigens as vaccine candidates such as merozoite or sporozoite surface proteins. However, the success has been limited thus far.

More recently, through the use of new technologies, immunoscreens have become more comprehensive, and has revealed the strong association of non-immunodominant malarial antigens with protection. Antigen libraries, expressed in bacterial, mammalian, or wheat germ cell-free expression systems, are created either as DNA libraries, recombinant proteins or on cell surface as antigen-presenting cells. In the screening of these antigen libraries for protective antigens, immune sera are an important tool. The type of immune sera chosen for the screening is critical. Carefully planned vaccination trials with an experimental challenge provide differential groups of sera from protected versus non-protected vaccinated individuals to identify protective antigens. Sera from chronically exposed individuals living in malaria-endemic regions have been also used in studies to screen for protective antigens. While sera from these individuals inform about naturally acquired immunity, it is essential to differentiate between the susceptible and the resistant individuals in these naturally exposed individuals. These differential sera allow the exclusion of immunodominant antigens, and inclusion of the non-immunodominant antigens that are associated with protection.

Taken together, the current consensus is protection against malaria is attributed to robust humoral responses directed against a panel of various non-variant antigens instead of only a single or a few immunodominant antigens. Studies to validate the feasibility of these minor non-immunodominant antigens as vaccine candidates should be prioritized for vaccine development against malarial infections. In addition, understanding antigen recognition is an essential step for the establishment of key immune correlates of protection against malarial infections, which would aid greatly in validating vaccine efficacy.

## Conflict of Interest Statement

The authors declare that the research was conducted in the absence of any commercial or financial relationships that could be construed as a potential conflict of interest.

## References

[B1] Agnandji S. T. Lell B. Fernandes J. F. Abossolo B. P. Methogo B. G. Kabwende A. L. (2012). A phase 3 trial of RTS,S/AS01 malaria vaccine in African infants. *N. Engl. J. Med.* 367 2284–2295 10.1056/NEJMoa1208394 23136909 PMC10915853

[B2] Agnandji S. T. Lell B. Soulanoudjingar S. S. Fernandes J. F. Abossolo B. P. Conzelmann C. (2011). First results of phase 3 trial of RTS,S/AS01 malaria vaccine in African children. *N. Engl. J. Med.* 365 1863–1875 10.1056/NEJMoa1102287 22007715

[B3] Amino R. Thiberge S. Martin B. Celli S. Shorte S. Frischknecht F. (2006). Quantitative imaging of *Plasmodium* transmission from mosquito to mammal. *Nat. Med.* 12 220–224 10.1038/nm1350 16429144

[B4] Anders R. F. (1986). Multiple cross-reactivities amongst antigens of *Plasmodium falciparum* impair the development of protective immunity against malaria. *Parasite Immunol.* 8 529–539 10.1111/j.1365-3024.1986.tb00867.x 3543808

[B5] Anders R. F. Coppel R. L. Brown G. V. Saint R. B. Cowman A. F. Lingelbach K. R. (1984). *Plasmodium falciparum* complementary DNA clones expressed in *Escherichia coli* encode many distinct antigens. *Mol. Biol. Med.* 2 177–191. 6399547

[B6] Ardeshir F. Flint J. E. Reese R. T. (1985). Expression of *Plasmodium falciparum* surface antigens in *Escherichia coli*. *Proc. Natl. Acad. Sci. U.S.A.* 82 2518–2522 10.1073/pnas.82.8.2518 3887406 PMC397590

[B7] Ashley E. A. Dhorda M. Fairhurst R. M. Amaratunga C. Lim P. Suon S. (2014). Spread of artemisinin resistance in *Plasmodium falciparum* malaria. *N. Engl. J. Med.* 371 411–423 10.1056/NEJMoa1314981 25075834 PMC4143591

[B8] Baer K. Klotz C. Kappe S. H. Schnieder T. Frevert U. (2007). Release of hepatic *Plasmodium yoelii* merozoites into the pulmonary microvasculature. *PLoS Pathog.* 3:e171 10.1371/journal.ppat.0030171 PMC2065874 17997605

[B9] Barry A. E. Trieu A. Fowkes F. J. Pablo J. Kalantari-Dehaghi M. Jasinskas A. (2011). The stability and complexity of antibody responses to the major surface antigen of *Plasmodium falciparum* are associated with age in a malaria endemic area. *Mol. Cell. Proteomics* 10:M111 10.1074/mcp.M111.008326 PMC3226400 21825279

[B10] Baum E. Badu K. Molina D. M. Liang X. Felgner P. L. Yan G. (2013). Protein microarray analysis of antibody responses to *Plasmodium falciparum* in western Kenyan highland sites with differing transmission levels. *PLoS ONE* 8:e82246 10.1371/journal.pone.0082246 PMC3846730 24312649

[B11] Bejon P. White M. T. Olotu A. Bojang K. Lusingu J. P. Salim N. (2013). Efficacy of RTS,S malaria vaccines: individual-participant pooled analysis of phase 2 data. *Lancet Infect. Dis.* 13 319–327 10.1016/S1473-3099(13)70005-7 23454164 PMC3771416

[B12] Belnoue E. Kayibanda M. Vigario A. M. Deschemin J. C. Van Rooijen N. Viguier M. (2002). On the pathogenic role of brain-sequestered alphabeta CD8+ T cells in experimental cerebral malaria. *J. Immunol.* 169 6369–6375 10.4049/jimmunol.169.11.6369 12444144

[B13] Belnoue E. Voza T. Costa F. T. Gruner A. C. Mauduit M. Rosa D. S. (2008). Vaccination with live *Plasmodium yoelii* blood stage parasites under chloroquine cover induces cross-stage immunity against malaria liver stage. *J. Immunol.* 181 8552–8558 10.4049/jimmunol.181.12.8552 19050274 PMC5551044

[B14] Bottius E. Benmohamed L. Brahimi K. Gras H. Lepers J. P. Raharimalala L. (1996). A novel *Plasmodium falciparum* sporozoite and liver stage antigen (SALSA) defines major B, T helper, and CTL epitopes. *J. Immunol.* 156 2874–2884. 8609407

[B15] Bouharoun-Tayoun H. Oeuvray C. Lunel F. Druilhe P. (1995). Mechanisms underlying the monocyte-mediated antibody-dependent killing of *Plasmodium falciparum* asexual blood stages. *J. Exp. Med.* 182 409–418 10.1084/jem.182.2.409 7629503 PMC2192140

[B16] Bozdech Z. Llinas M. Pulliam B. L. Wong E. D. Zhu J. Derisi J. L. (2003). The transcriptome of the intraerythrocytic developmental cycle of *Plasmodium falciparum*. *PLoS Biol.* 1:E5 10.1371/journal.pbio.0000005 PMC176545 12929205

[B17] Brown G. V. Anders R. F. Coppel R. L. Saint R. B. Cowman A. F. Stahl H. D. (1984). The expression of *Plasmodium falciparum* bloodstage antigens in *Escherichia coli*. *Philos. Trans. R. Soc. Lond. B Biol. Sci.* 307 179–187 10.1098/rstb.1984.0118 6151682

[B18] Butler N. S. Vaughan A. M. Harty J. T. Kappe S. H. (2012). Whole parasite vaccination approaches for prevention of malaria infection. *Trends Immunol.* 33 247–254 10.1016/j.it.2012.02.001 22405559

[B19] Carlton J. M. Angiuoli S. V. Suh B. B. Kooij T. W. Pertea M. Silva J. C. (2002). Genome sequence and comparative analysis of the model rodent malaria parasite *Plasmodium yoelii* yoelii. *Nature* 419 512–519 10.1038/nature01099 12368865

[B20] CDC (2012). *Impact of Malaria [Online].* Available at: http://www.cdc.gov/malaria/malaria_worldwide/impact.html [Accessed Feb 14 2014].

[B21] Chia W. Asm O. Ksw T. Preiser P. Renia L. (2014). “Defining immune correlates of protection against malaria using *Plasmodium yoelii* mouse models,” in *Australian Society of Parasitology Annual Conference* Canberra.

[B22] Clyde D. F. Most H. Mccarthy V. C. Vanderberg J. P. (1973). Immunization of man against sporozite-induced falciparum malaria. *Am. J. Med. Sci.* 266 169–177 10.1097/00000441-197309000-00002 4583408

[B23] Cockburn I. A. Amino R. Kelemen R. K. Kuo S. C. Tse S. W. Radtke A. (2013). In vivo imaging of CD8+ T cell-mediated elimination of malaria liver stages. *Proc. Natl. Acad. Sci. U.S.A.* 110 9090–9095 10.1073/pnas.1303858110 23674673 PMC3670364

[B24] Cohen S. Mc G. I. Carrington S. (1961). Gamma-globulin and acquired immunity to human malaria. *Nature* 192 733–737 10.1038/192733a0 13880318

[B25] Crompton P. D. Kayala M. A. Traore B. Kayentao K. Ongoiba A. Weiss G. E. (2010). A prospective analysis of the Ab response to *Plasmodium falciparum* before and after a malaria season by protein microarray. *Proc. Natl. Acad. Sci. U.S.A.* 107 6958–6963 10.1073/pnas.1001323107 20351286 PMC2872454

[B26] Crosnier C. Bustamante L. Y. Bartholdson S. J. Bei A. K. Theron M. Uchikawa M. (2011). Basigin is a receptor essential for erythrocyte invasion by *Plasmodium falciparum*. *Nature* 480 534–537 10.1038/nature10606 22080952 PMC3245779

[B27] Daubersies P. Thomas A. W. Millet P. Brahimi K. Langermans J. A. Ollomo B. (2000). Protection against *Plasmodium falciparum* malaria in chimpanzees by immunization with the conserved pre-erythrocytic liver-stage antigen 3. *Nat. Med.* 6 1258–1263 10.1038/81366 11062538

[B28] Doolan D. L. (2011). *Plasmodium* immunomics. *Int. J. Parasitol.* 41 3–20 10.1016/j.ijpara.2010.08.002 20816843 PMC3005034

[B29] Doolan D. L. Hoffman S. L. (2000). The complexity of protective immunity against liver-stage malaria. *J. Immunol.* 165 1453–1462 10.4049/jimmunol.165.3.1453 10903750

[B30] Doolan D. L. Mu Y. Unal B. Sundaresh S. Hirst S. Valdez C. (2008). Profiling humoral immune responses to *Plasmodium falciparum* infection with protein microarrays. *Proteomics* 8 4680–4694 10.1002/pmic.200800194 18937256 PMC3021802

[B31] Doolan D. L. Southwood S. Freilich D. A. Sidney J. Graber N. L. Shatney L. (2003). Identification of *Plasmodium falciparum* antigens by antigenic analysis of genomic and proteomic data. *Proc. Natl. Acad. Sci. U.S.A.* 100 9952–9957 10.1073/pnas.1633254100 12886016 PMC187898

[B32] Dutta S. Haynes J. D. Barbosa A. Ware L. A. Snavely J. D. Moch J. K. (2005). Mode of action of invasion-inhibitory antibodies directed against apical membrane antigen 1 of *Plasmodium falciparum*. *Infect. Immun.* 73 2116–2122 10.1128/IAI.73.4.2116-2122.2005 15784553 PMC1087451

[B33] Ellis J. Ozaki L. S. Gwadz R. W. Cochrane A. H. Nussenzweig V. Nussenzweig R. S. (1983). Cloning and expression in *E. coli* of the malarial sporozoite surface antigen gene from *Plasmodium knowlesi. Nature* 302 536–538 10.1038/302536a0 6339949

[B34] Fidock D. A. Sallenave-Sales S. Sherwood J. A. Gachihi G. S. Ferreira-Da-Cruz M. F. Thomas A. W. (1994). Conservation of the *Plasmodium falciparum* sporozoite surface protein gene, STARP, in field isolates and distinct species of *Plasmodium*. *Mol. Biochem. Parasitol.* 67 255–267 10.1016/0166-6851(94)00138-3 7870130

[B35] Finney O. C. Keitany G. J. Smithers H. Kaushansky A. Kappe S. Wang R. (2014). Immunization with genetically attenuated *Plasmodium falciparum* parasites induces long-lived antibodies that efficiently block hepatocyte invasion by sporozoites. *Vaccine* 32 2135–2138 10.1016/j.vaccine.2014.02.055 24582635 PMC4337823

[B36] Florens L. Washburn M. P. Raine J. D. Anthony R. M. Grainger M. Haynes J. D. (2002). A proteomic view of the *Plasmodium falciparum* life cycle. *Nature* 419 520–526 10.1038/nature01107 12368866

[B37] Frevert U. Moreno A. Calvo-Calle M. Klotz C. Nardin E. H. (2009). Imaging effector functions of human cytotoxic CD4+ T cells specific for *Plasmodium falciparum* circumsporozoite protein. *Int. J. Parasitol.* 39 119–132 10.1016/j.ijpara.2008.06.014 18723023 PMC3021960

[B38] Friesen J. Silvie O. Putrianti E. D. Hafalla J. C. Matuschewski K. Borrmann S. (2010). Natural immunization against malaria: causal prophylaxis with antibiotics. *Sci. Transl. Med.* 2 40–49 10.1126/scitranslmed.3001058 20630856

[B39] Gardner M. J. Hall N. Fung E. White O. Berriman M. Hyman R. W. (2002). Genome sequence of the human malaria parasite *Plasmodium falciparum*. *Nature* 419 498–511 10.1038/nature01097 12368864 PMC3836256

[B40] Gruner A. C. Brahimi K. Eling W. Konings R. Meis J. Aikawa M. (2001a). The *Plasmodium falciparum* knob-associated PfEMP3 antigen is also expressed at pre-erythrocytic stages and induces antibodies which inhibit sporozoite invasion. *Mol. Biochem. Parasitol.* 112 253–261 10.1016/S0166-6851(00)00373-X 11223132

[B41] Gruner A. C. Brahimi K. Letourneur F. Renia L. Eling W. Snounou G. (2001b). Expression of the erythrocyte-binding antigen 175 in sporozoites and in liver stages of *Plasmodium falciparum*. *J. Infect. Dis.* 184 892–897 10.1086/323394 11528591

[B42] Gruner A. C. Snounou G. Brahimi K. Letourneur F. Renia L. Druilhe P. (2003). Pre-erythrocytic antigens of *Plasmodium falciparum*: from rags to riches? *Trends Parasitol.* 19 74–78 10.1016/j.ijpara.2004.05.005 12586475

[B43] Guerin-Marchand C. Druilhe P. Galey B. Londono A. Patarapotikul J. Beaudoin R. L. (1987). A liver-stage-specific antigen of *Plasmodium falciparum* characterized by gene cloning. *Nature* 329 164–167 10.1038/329164a0 3306406

[B44] Gupta S. Snow R. W. Donnelly C. A. Marsh K. Newbold C. (1999). Immunity to non-cerebral severe malaria is acquired after one or two infections. *Nat. Med.* 5 340–343 10.1038/6560 10086393

[B45] Haddad D. Bilcikova E. Witney A. A. Carlton J. M. White C. E. Blair P. L. (2004). Novel antigen identification method for discovery of protective malaria antigens by rapid testing of DNA vaccines encoding exons from the parasite genome. *Infect. Immun.* 72 1594–1602 10.1128/IAI.72.3.1594-1602.2004 14977966 PMC356014

[B46] Hall N. Karras M. Raine J. D. Carlton J. M. Kooij T. W. Berriman M. (2005). A comprehensive survey of the *Plasmodium* life cycle by genomic, transcriptomic, and proteomic analyses. *Science* 307 82–86 10.1126/science.1103717 15637271

[B47] Hall N. Pain A. Berriman M. Churcher C. Harris B. Harris D. (2002). Sequence of *Plasmodium falciparum* chromosomes 1 3–9 and 13. *Nature* 419 527–531 10.1038/nature01095 12368867

[B48] Hoffman S. L. Goh L. M. Luke T. C. Schneider I. Le T. P. Doolan D. L. (2002). Protection of humans against malaria by immunization with radiation-attenuated *Plasmodium falciparum* sporozoites. *J. Infect. Dis.* 185 1155–1164 10.1086/339409 11930326

[B49] Horne-Debets J. M. Faleiro R. Karunarathne D. S. Liu X. Q. Lineburg K. E. Poh C. M. (2013). PD-1 dependent exhaustion of CD8+ T cells drives chronic malaria. *Cell Rep.* 5 1204–1213 10.1016/j.celrep.2013.11.002 24316071

[B50] Jiang L. Gaur D. Mu J. Zhou H. Long C. A. Miller L. H. (2011). Evidence for erythrocyte-binding antigen 175 as a component of a ligand-blocking blood-stage malaria vaccine. *Proc. Natl. Acad. Sci. U.S.A.* 108 7553–7558 10.1073/pnas.1104050108 21502513 PMC3088598

[B51] Kamali A. N. Marin-Garcia P. Azcarate I. G. Diez A. Puyet A. Bautista J. M. (2012). *Plasmodium yoelii* blood-stage antigens newly identified by immunoaffinity using purified IgG antibodies from malaria-resistant mice. *Immunobiology* 217 823–830 10.1016/j.imbio.2012.05.002 22658767

[B52] Kappe S. H. Gardner M. J. Brown S. M. Ross J. Matuschewski K. Ribeiro J. M. (2001). Exploring the transcriptome of the malaria sporozoite stage. *Proc. Natl. Acad. Sci. U.S.A.* 98 9895–9900 10.1073/pnas.171185198 11493695 PMC55549

[B53] Kappe S. H. Noe A. R. Fraser T. S. Blair P. L. Adams J. H. (1998). A family of chimeric erythrocyte binding proteins of malaria parasites. *Proc. Natl. Acad. Sci. U.S.A.* 95 1230–1235 10.1073/pnas.95.3.1230 9448314 PMC18728

[B54] Kemp D. J. Coppel R. L. Anders R. F. (1987). Repetitive proteins and genes of malaria. *Annu. Rev. Microbiol.* 41 181–208 10.1146/annurev.mi.41.100187.001145 3318667

[B55] Kemp D. J. Coppel R. L. Cowman A. F. Saint R. B. Brown G. V. Anders R. F. (1983). Expression of *Plasmodium falciparum* blood-stage antigens in *Escherichia coli*: detection with antibodies from immune humans. *Proc. Natl. Acad. Sci. U.S.A.* 80 3787–3791 10.1073/pnas.80.12.3787 6304737 PMC394137

[B56] Kemp D. J. Coppel R. L. Stahl H. D. Bianco A. E. Corcoran L. M. Mcintyre P. (1986). The wellcome trust lecture. Genes for antigens of *Plasmodium falciparum. Parasitology* 92(Suppl.) S83–S108 10.1017/S0031182000085711 2423947

[B57] Khan S. M. Franke-Fayard B. Mair G. R. Lasonder E. Janse C. J. Mann M. (2005). Proteome analysis of separated male and female gametocytes reveals novel sex-specific *Plasmodium* biology. *Cell* 121 675–687 10.1016/j.cell.2005.03.027 15935755

[B58] Kooij T. W. Carlton J. M. R. Bidwell S. L. Hall N. Ramesar J. Janse C. J. (2005). A *Plasmodium* whole-genome synteny map: indels and synteny breakpoints as foci for species-specific genes. *PLoS Pathog.* 1:e44 10.1371/journal.ppat.0010044 PMC1317653 16389297

[B59] Langhorne J. (1994). The immune response to the blood stages of *Plasmodium* in animal models. *Immunol. Lett.* 41 99–102 10.1016/0165-2478(94)90115-5 8002055

[B60] Langhorne J. Ndungu F. M. Sponaas A. M. Marsh K. (2008). Immunity to malaria: more questions than answers. *Nat. Immunol.* 9 725–732 10.1038/ni.f.205 18563083

[B61] Lasonder E. Ishihama Y. Andersen J. S. Vermunt A. M. Pain A. Sauerwein R. W. (2002). Analysis of the *Plasmodium falciparum* proteome by high-accuracy mass spectrometry. *Nature* 419 537–542 10.1038/nature01111 12368870

[B62] Le Roch K. G. Zhou Y. Batalov S. Winzeler E. A. (2002). Monitoring the chromosome 2 intraerythrocytic transcriptome of *Plasmodium falciparum* using oligonucleotide arrays. *Am. J. Trop. Med. Hyg.* 67 233–243. 12408661 10.4269/ajtmh.2002.67.233

[B63] Lindner S. E. Swearingen K. E. Harupa A. Vaughan A. M. Sinnis P. Moritz R. L. (2013). Total and putative surface proteomics of malaria parasite salivary gland sporozoites. *Mol. Cell. Proteomics* 12 1127–1143 10.1074/mcp.M112.024505 23325771 PMC3650326

[B64] Lu F. Li J. Wang B. Cheng Y. Kong D. H. Cui L. (2014). Profiling the humoral immune responses to *Plasmodium vivax* infection and identification of candidate immunogenic rhoptry-associated membrane antigen (RAMA). *J. Proteomics* 6 66–82 10.1016/j.jprot.2014.02.029 24607491

[B65] Marchand C. Druilhe P. (1990). How to select *Plasmodium falciparum* pre-erythrocytic antigens in an expression library without defined probe. *Bull. World Health Organ.* 68(Suppl.) 158–164. 1709833 PMC2393044

[B66] Marsh K. Kinyanjui S. (2006). Immune effector mechanisms in malaria. *Parasite Immunol.* 28 51–60 10.1111/j.1365-3024.2006.00808.x 16438676

[B67] Mehlin C. Boni E. Buckner F. S. Engel L. Feist T. Gelb M. H. (2006). Heterologous expression of proteins from *Plasmodium falciparum*: results from 1000 genes. *Mol. Biochem. Parasitol.* 148 144–160 10.1016/j.molbiopara.2006.03.011 16644028

[B68] Michon P. Fraser T. Adams J. H. (2000). Naturally acquired and vaccine-elicited antibodies block erythrocyte cytoadherence of the *Plasmodium vivax* Duffy binding protein. *Infect. Immun.* 68 3164–3171 10.1128/IAI.68.6.3164-3171.2000 10816459 PMC97553

[B69] Molina D. M. Finney O. C. Arevalo-Herrera M. Herrera S. Felgner P. L. Gardner M. J. (2012). *Plasmodium vivax* pre-erythrocytic-stage antigen discovery: exploiting naturally acquired humoral responses. *Am. J. Trop. Med. Hyg.* 87 460–469 10.4269/ajtmh.2012.12-0222 22826492 PMC3435348

[B70] Moorthy V. S. Good M. F. Hill A. V. (2004). Malaria vaccine developments. *Lancet* 363 150–156 10.1016/S0140-6736(03)15267-1 14726170

[B71] Mueller A. K. Labaied M. Kappe S. H. Matuschewski K. (2005). Genetically modified *Plasmodium* parasites as a protective experimental malaria vaccine. *Nature* 433 164–167 10.1038/nature03188 15580261

[B72] Nussenzweig R. S. Vanderberg J. Most H. Orton C. (1967). Protective immunity produced by the injection of x-irradiated sporozoites of *Plasmodium berghei*. *Nature* 216 160–162 10.1038/216160a0 6057225

[B73] Offeddu V. Thathy V. Marsh K. Matuschewski K. (2012). Naturally acquired immune responses against *Plasmodium falciparum* sporozoites and liver infection. *Int. J. Parasitol.* 42 535–548 10.1016/j.ijpara.2012.03.011 22561398

[B74] Olotu A. Fegan G. Wambua J. Nyangweso G. Awuondo K. O. Leach A. (2013). Four-year efficacy of RTS,S/AS01E and its interaction with malaria exposure. *N. Engl. J. Med.* 368 1111–1120 10.1056/NEJMoa1207564 23514288 PMC5156295

[B75] Osier F. H. Mackinnon M. J. Crosnier C. Fegan G. Kamuyu G. Wanaguru M. (2014). New antigens for a multicomponent blood-stage malaria vaccine. *Sci. Transl. Med.* 6 247–102 10.1126/scitranslmed.3008705 PMC4856877 25080477

[B76] Overstreet M. G. Cockburn I. A. Chen Y. C. Zavala F. (2008). Protective CD8 T cells against *Plasmodium* liver stages: immunobiology of an ‘unnatural’ immune response. *Immunol. Rev.* 225 272–283 10.1111/j.1600-065X.2008.00671.x 18837788 PMC2597001

[B77] Phyo A. P. Nkhoma S. Stepniewska K. Ashley E. A. Nair S. Mcgready R. (2012). Emergence of artemisinin-resistant malaria on the western border of Thailand: a longitudinal study. *Lancet* 379 1960–1966 10.1016/S0140-6736(12)60484-X 22484134 PMC3525980

[B78] Raj D. K. Nixon C. P. Nixon C. E. Dvorin J. D. Dipetrillo C. G. Pond-Tor S. (2014). Antibodies to PfSEA-1 block parasite egress from RBCs and protect against malaria infection. *Science* 344 871–877 10.1126/science.1254417 24855263 PMC4184151

[B79] Rathore D. Nagarkatti R. Jani D. Chattopadhyay R. De La Vega P. Kumar S. (2005). An immunologically cryptic epitope of *Plasmodium falciparum* circumsporozoite protein facilitates liver cell recognition and induces protective antibodies that block liver cell invasion. *J. Biol. Chem.* 280 20524–20529 10.1074/jbc.M414254200 15781464

[B80] Renia L. Grillot D. Marussig M. Corradin G. Miltgen F. Lambert P. H. (1993). Effector functions of circumsporozoite peptide-primed CD4+ T cell clones against *Plasmodium yoelii* liver stages. *J. Immunol.* 150 1471–1478. 7679428

[B81] Renia L. Gruner A. C. Mauduit M. Snounou G. (2006). Vaccination against malaria with live parasites. *Expert Rev. Vaccines* 5 473–481 10.1586/14760584.5.4.473 16989628

[B82] Renia L. Marussig M. S. Grillot D. Pied S. Corradin G. Miltgen F. (1991). In vitro activity of CD4+ and CD8+ T lymphocytes from mice immunized with a synthetic malaria peptide. *Proc. Natl. Acad. Sci. U.S.A.* 88 7963–7967 10.1073/pnas.88.18.7963 1680235 PMC52425

[B83] Renia L. Mattei D. Goma J. Pied S. Dubois P. Miltgen F. (1990). A malaria heat-shock-like determinant expressed on the infected hepatocyte surface is the target of antibody-dependent cell-mediated cytotoxic mechanisms by nonparenchymal liver cells. *Eur. J. Immunol.* 20 1445–1449 10.1002/eji.1830200706 2201546

[B84] Richards J. S. Arumugam T. U. Reiling L. Healer J. Hodder A. N. Fowkes F. J. (2013). Identification and prioritization of merozoite antigens as targets of protective human immunity to *Plasmodium falciparum* malaria for vaccine and biomarker development. *J. Immunol.* 191 795–809 10.4049/jimmunol.1300778 23776179 PMC3702023

[B85] Richards W. H. (1966). Antimalarial activity of sulphonamides and a sulphone, singly and in combination with pyrimethamine, against drug resistant and normal strains of laboratory plasmodia. *Nature* 212 1494–1495 10.1038/2121494a0 21090431

[B86] Rieckmann K. H. Carson P. E. Beaudoin R. L. Cassells J. S. Sell K. W. (1974). Letter: sporozoite induced immunity in man against an Ethiopian strain of *Plasmodium falciparum*. *Trans. R. Soc. Trop. Med. Hyg.* 68 258–259 10.1016/0035-9203(74)90129-1 4608063

[B87] Robson K. J. Frevert U. Reckmann I. Cowan G. Beier J. Scragg I. G. (1995). Thrombospondin-related adhesive protein (TRAP) of *Plasmodium falciparum*: expression during sporozoite ontogeny and binding to human hepatocytes. *EMBO J.* 14 3883–3894. 7664729 10.1002/j.1460-2075.1995.tb00060.x PMC394467

[B88] Rodriguez L. E. Curtidor H. Urquiza M. Cifuentes G. Reyes C. Patarroyo M. E. (2008). Intimate molecular interactions of *Plasmodium falciparum* merozoite proteins involved in invasion of red blood cells and their implications for vaccine design. *Chem. Rev.* 108 3656–3705 10.1021/cr068407v 18710292

[B89] Roestenberg M. Mccall M. Hopman J. Wiersma J. Luty A. J. Van Gemert G. J. (2009). Protection against a malaria challenge by sporozoite inoculation. *N. Engl. J. Med.* 361 468–477 10.1056/NEJMoa0805832 19641203

[B90] Rui E. Fernandez-Becerra C. Takeo S. Sanz S. Lacerda M. V. Tsuboi T. (2011). *Plasmodium vivax*: comparison of immunogenicity among proteins expressed in the cell-free systems of *Escherichia coli* and wheat germ by suspension array assays. *Malar. J.* 10 192 10.1186/1475-2875-10-192 PMC3224337 21756349

[B91] Schofield L. Mueller I. (2006). Clinical immunity to malaria. *Curr. Mol. Med.* 6 205–221 10.2174/156652406776055221 16515511

[B92] Seder R. A. Chang L. J. Enama M. E. Zephir K. L. Sarwar U. N. Gordon I. J. (2013). Protection against malaria by intravenous immunization with a nonreplicating sporozoite vaccine. *Science* 341 1359–1365 10.1126/science.1241800 23929949

[B93] Sheehy S. H. Duncan C. J. Elias S. C. Choudhary P. Biswas S. Halstead F. D. (2012). ChAd63-MVA-vectored blood-stage malaria vaccines targeting MSP1 and AMA1: assessment of efficacy against mosquito bite challenge in humans. *Mol. Ther.* 20 2355–2368 10.1038/mt.2012.223 23089736 PMC3519995

[B94] Silvie O. Franetich J. F. Charrin S. Mueller M. S. Siau A. Bodescot M. (2004). A role for apical membrane antigen 1 during invasion of hepatocytes by *Plasmodium falciparum* sporozoites. *J. Biol. Chem.* 279 9490–9496 10.1074/jbc.M311331200 14676185

[B95] Singh N. Preiser P. Renia L. Balu B. Barnwell J. Blair P. (2004). Conservation and developmental control of alternative splicing in maebl among malaria parasites. *J. Mol. Biol.* 343 589–599 10.1016/j.jmb.2004.08.047 15465047

[B96] Spring M. Murphy J. Nielsen R. Dowler M. Bennett J. W. Zarling S. (2013). First-in-human evaluation of genetically attenuated *Plasmodium falciparum* sporozoites administered by bite of *Anopheles* mosquitoes to adult volunteers. *Vaccine* 31 4975–4983 10.1016/j.vaccine.2013.08.007 24029408

[B97] Spring M. D. Cummings J. F. Ockenhouse C. F. Dutta S. Reidler R. Angov E. (2009). Phase 1/2a study of the malaria vaccine candidate apical membrane antigen-1 (AMA-1) administered in adjuvant system AS01B or AS02A. *PLoS ONE* 4:e5254 10.1371/journal.pone.0005254 PMC2669163 19390585

[B98] Stahl H. D. Coppel R. L. Brown G. V. Saint R. Lingelbach K. Cowman A. F. (1984). Differential antibody screening of cloned *Plasmodium falciparum* sequences expressed in *Escherichia coli*: procedure for isolation of defined antigens and analysis of human antisera. *Proc. Natl. Acad. Sci. U.S.A.* 81 2456–2460 10.1073/pnas.81.8.2456 6371814 PMC345080

[B99] Stevenson M. M. Riley E. M. (2004). Innate immunity to malaria. *Nat. Rev. Immunol.* 4 169–180 10.1038/nri1311 15039754

[B100] Sturm A. Amino R. Van De Sand C. Regen T. Retzlaff S. Rennenberg A. (2006). Manipulation of host hepatocytes by the malaria parasite for delivery into liver sinusoids. *Science* 313 1287–1290 10.1126/science.1129720 16888102

[B101] Tachibana S. Sullivan S. A. Kawai S. Nakamura S. Kim H. R. Goto N. (2012). *Plasmodium* cynomolgi genome sequences provide insight into *Plasmodium vivax* and the monkey malaria clade. *Nat. Genet.* 44 1051–1055 10.1038/ng.2375 22863735 PMC3759362

[B102] Tarun A. S. Peng X. Dumpit R. F. Ogata Y. Silva-Rivera H. Camargo N. (2008). A combined transcriptome and proteome survey of malaria parasite liver stages. *Proc. Natl. Acad. Sci. U.S.A.* 105 305–310 10.1073/pnas.0710780104 18172196 PMC2224207

[B103] Taylor-Robinson A. W. (2010). Regulation of immunity to *Plasmodium*: implications from mouse models for blood stage malaria vaccine design. *Exp. Parasitol.* 126 406–414 10.1016/j.exppara.2010.01.028 20138874

[B104] Trape J. F. Tall A. Diagne N. Ndiath O. Ly A. B. Faye J. (2011). Malaria morbidity and pyrethroid resistance after the introduction of insecticide-treated bednets and artemisinin-based combination therapies: a longitudinal study. *Lancet Infect. Dis.* 11 925–932 10.1016/S1473-3099(11)70194-3 21856232

[B105] Trieu A. Kayala M. A. Burk C. Molina D. M. Freilich D. A. Richie T. L. (2011). Sterile protective immunity to malaria is associated with a panel of novel *Plasmodium falciparum* antigens. *Mol. Cell. Proteomics* 10 M111 007948. 10.1074/mcp.M111.007948 PMC3186199 21628511

[B106] Trimnell A. Takagi A. Gupta M. Richie T. L. Kappe S. H. Wang R. (2009). Genetically attenuated parasite vaccines induce contact-dependent CD8+ T cell killing of *Plasmodium yoelii* liver stage-infected hepatocytes. *J. Immunol.* 183 5870–5878 10.4049/jimmunol.0900302 19812194

[B107] Tsuboi T. Takeo S. Iriko H. Jin L. Tsuchimochi M. Matsuda S. (2008). Wheat germ cell-free system-based production of malaria proteins for discovery of novel vaccine candidates. *Infect. Immun.* 76 1702–1708 10.1128/IAI.01539-07 18268027 PMC2292889

[B108] Vanderberg J. P. Frevert U. (2004). Intravital microscopy demonstrating antibody-mediated immobilization of *Plasmodium berghei* sporozoites injected into skin by mosquitoes. *Int. J. Parasitol.* 34 991–996 10.1016/j.ijpara.2004.05.005 15313126

[B109] WHO (2012). *Malaria Vaccine Rainbow Tables.* Available at: http://www.who.int/vaccine_research/links/Rainbow/en/index.html [accessed June 30 2014].

[B110] Wykes M. N. Good M. F. (2009). What have we learnt from mouse models for the study of malaria? *Eur. J. Immunol.* 39 2004–2007 10.1002/eji.200939552 19672886

